# HIV Protein Nef Induces Cardiomyopathy Through Induction of Bcl2 and p21

**DOI:** 10.3390/ijms252111401

**Published:** 2024-10-23

**Authors:** Olena Kondrachuk, Pierce Ciccone, Nicole Ford, Kim Hong, Yuka Kimura, Jorgo Zi, Sumaya Yusuf, Aya Alkousa, Nishit Tailor, Rithvik Rajkumar, Jay Rappaport, Manish K. Gupta

**Affiliations:** 1 Division of Metabolic and Cardiovascular Sciences, Burnett School of Biomedical Sciences, College of Medicine, University of Central Florida, Orlando, FL 32827, USA; 2 Division of Pathology, Tulane National Primate Research Center, Covington, LA 70118, USA

**Keywords:** HIV, Nef, autophagy, cardiomyopathy, Bcl2, Beclin-1, fibrosis, P21

## Abstract

HIV-associated cardiovascular diseases remain a leading cause of death in people living with HIV/AIDS (PLWHA). Although antiretroviral drugs suppress the viral load, they fail to remove the virus entirely. HIV-1 Nef protein is known to play a role in viral virulence and HIV latency. Expression of Nef protein can be detected in different organs, including cardiac tissue. Despite the established role of Nef protein in HIV-1 replication, its impact on organ function inside the human body is not clear. To understand the effect of Nef at the organ level, we created a new Nef-transgenic (Nef-TG) mouse that expresses Nef protein in the heart. Our study found that Nef expression caused inhibition of cardiac function and pathological changes in the heart with increased fibrosis, leading to heart failure and early mortality. Further, we found that cellular autophagy is significantly inhibited in the cardiac tissue of Nef-TG mice. Mechanistically, we found that Nef protein causes the accumulation of Bcl2 and Beclin-1 proteins in the tissue, which may affect the cellular autophagy system. Additionally, we found Nef expression causes upregulation of the cellular senescence marker p21 and senescence-associated β-galactosidase expression. Our findings suggest that the Nef-mediated inhibition of autophagy and induction of senescence markers may promote aging in PLWHA. Our mouse model could help us to understand the effect of Nef protein on organ function during latent HIV infection.

## 1. Introduction

According to the 22 July 2024 World Health Organization report, 39.9 million people were living with HIV/AIDS (PLWHA) globally in 2023, and 77% of those had access to antiretroviral therapy (ART) [1]. Application of current ART notably increases the life expectancy of HIV carriers, switching HIV disease from an acute to a chronic illness. However, recent studies suggest PLWHA develop several comorbidities, and cardiovascular disease remains one of the leading causes of death in HIV patients [2]. HIV-infected patients show several signs of cardiovascular disease, including myocardial infarctions, abnormalities in the aorta, aortic inflammation, and intramyocardial fibrosis, with an increased risk of sudden cardiac death compared to uninfected individuals [3]. Because modern ART regimes substantially decrease viral loads, the direct HIV impact on organ systems is diminished in people with adequate ART [4]. However, expression of HIV proteins can still be detected in different organs of ART-treated individuals, which may affect the organ’s function. Our lab and several other researchers have reported the presence of one of the HIV proteins, negative factor (Nef), in the heart of ART-treated HIV patients [5,6,7,8,9]. Depending upon the isoforms, Nef protein can be 27- to 35-kDa protein and post-translationally modified by myristylation [10]. Earlier studies suggest that Nef plays an important role in HIV-1 replication and pathogenesis [5,11]. Since the Nef protein is found in different organs of HIV patients, it may modulate the function of various organs through the induction of cellular toxicity [6,8,9,12]. Specifically, Nef regulates cellular autophagy [8]. Additionally, protein interaction studies show that Nef protein regulates autophagy steps through interaction with the autophagy regulatory proteins Beclin-1 and p62 [13,14]. Further, we found that Nef protein induces cardiotoxicity in cell culture models [8]. However, the role of Nef protein under in vivo conditions needs to be established.

To understand the effect of Nef protein on organ function, we have generated a new Nef-transgenic mice model. Functional study shows that Nef compromises heart function and induces end-stage heart failure. Further, our study found that Nef expression modulates the balance of key autophagy regulatory complex Bcl2 and Beclin-1. The dysregulation of Bcl2–Beclin-1 expression may inhibit autophagy and help HIV to sustain in the host cells. Moreover, we found that Nef protein promotes the senescence of cardiac cells by regulating the expression of cyclin-dependent kinase inhibitor p21 and senescence-associated β-galactosidase (SA-βgal) expression.

## 2. Results

### 2.1. Nef-Transgenic Mouse Was Generated to Express Nef Protein in an Organ-Specific Manner

Previous studies suggest that Nef proteins can be detected in different organs of HIV patients. To elucidate the role of Nef protein in HIV-mediated pathogenesis, we have generated a new transgenic mouse that can express Nef protein in different cell and tissue types in the presence of Cre recombinase. A full-length HIV-1 Nef ORF was cloned in a CAG-lox-CAT-lox vector to create Nef-transgenic mice, as described in Figure S1A. Several founder mice were generated. To check the expression of Nef protein in cardiac tissue, we crossed the founder mice lines with the α-MyHC-Cre mice lines. A Western blot was performed to check the Nef protein expression in the heart tissue of the Nef-positive mice. Interestingly, we can detect the Nef protein in transgenic mice positive for both the Nef and Cre genes (Figure S1B). Further, our analysis shows that Nef-transgenic mice lines have different levels of Nef protein expression (Figure S1C).

### 2.2. Nef-Transgenic Mice Develop Cardiac Abnormality and Fibrosis

**Figure 1:**
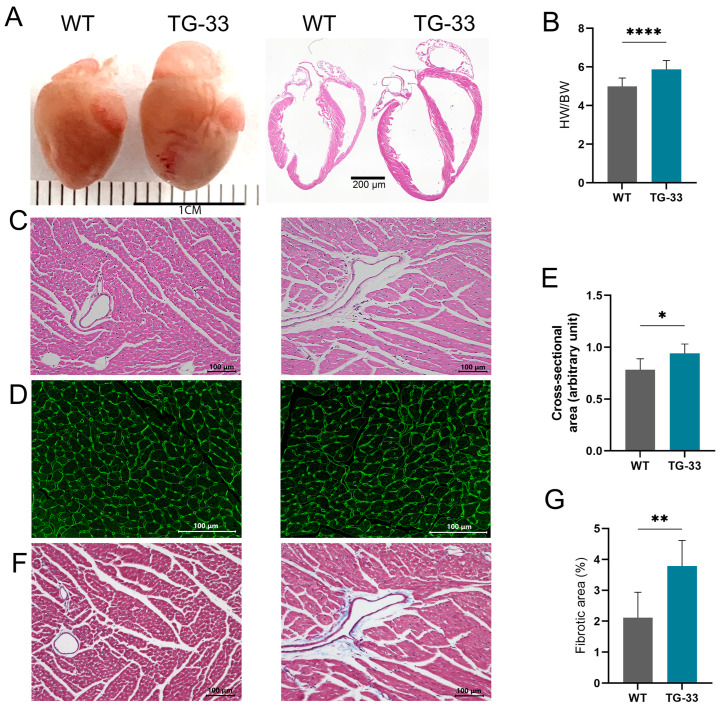
Nef protein expression alters cardiac morphology and induces fibrosis in the heart. For histological analysis, heart tissues from WT and TG-33 mice were collected at 10–13 weeks. (**A**) Representative images show whole heart and butterfly sections of the heart stained with H&E. (**B**) Graph shows the heart-to-body weight ratio (*n* = 38 WT (20 males, 18 females) and 11 TG-33 (5 males, 6 females)). Representative images show the paraffin tissue section stained with (**C**) H&E, (**D**) Masson’s trichrome, and (**F**) WGA stains. The graphs show quantification of (**E**) the cross-section area of cells and (**G**) the fibrotic area (*n* = 7 WT (5 males, 2 females) and 6 TG-33 (2 males, 4 females)). Data are presented with standard deviation. Statistical significances were calculated between WT and TG-33 mice (* *p* ≤ 0.05, ** *p* ≤ 0.01, **** *p* ≤ 0.0001).

To understand the impact of Nef protein on cardiac morphology and pathological changes, we examined the cardiac tissue of Nef-transgenic mice by histological staining. Fixed hearts were collected from the adult TG-33 mice and their wild-type (WT) littermates. Heart tissues were processed, and thin paraffin sections were prepared. Paraffin tissue sections were stained with H&E, WGA, and Masson’s trichrome staining to evaluate the role of Nef protein in cardiac remodeling. Gross morphological examination of heart sections stained with the H&E stain shows that TG-33 mice have an enlarged heart size compared to their wild-type littermates (Figure 1A). Furthermore, heart-to-body weight ratio analysis suggests that Nef-transgenic mice have cardiac hypertrophy (Figure 1B). The H&E staining of the heart tissue sections shows cardiomyocyte disarray and accumulation of immune cells in the TG-33 mice’s hearts (Figure 1C). We also performed cell size analysis by microscopy of WGA-stained paraffin tissue sections (Figure 1D). Cell size analysis further shows that Nef-transgenic mice have a larger cardiomyocyte area compared to their nontransgenic littermates (Figure 1E), which serves as an additional confirmation of cardiac hypertrophy. Moreover, we detected cardiac fibrosis in the Masson’s trichrome-stained tissue section (Figure 1F). Quantification of the fibrotic area shows that Nef-TG mice have a higher fibrotic area compared to their WT littermates (Figure 1G).

### 2.3. Nef Protein Expression Compromises Heart Function

**Figure 2:**
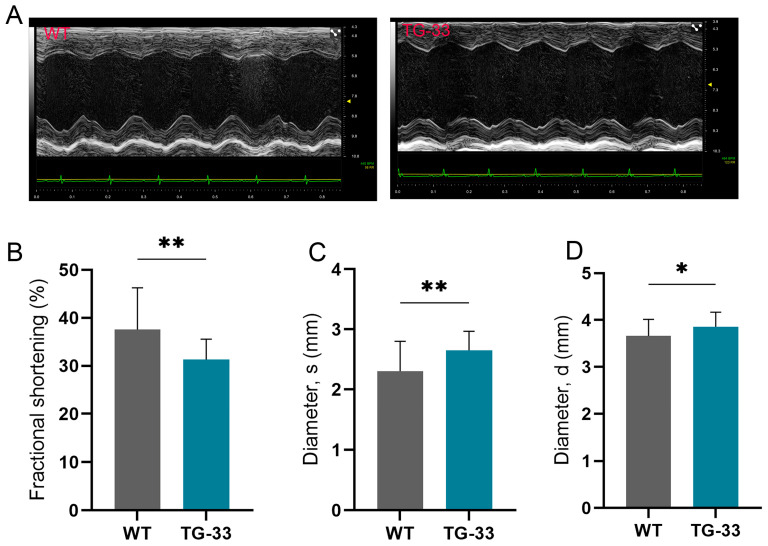
**Nef-transgenic mice exhibit cardiac dysfunction.** (**A**) Representative images show M-mode echocardiography of the wild-type (WT) and TG-33 mice at 10–13 weeks of age. The images of the left ventricle were captured at the mid-papillary level (the parasternal short-axis view). The graphs show quantification of (**B**) fractional shortening and (**C**) diameter of the left ventricle during systole (diameter, s) and (**D**) during diastole (diameter, d) (*n* = 32 WT (16 males, 16 females) and 24 TG-33 (13 males, 11 females)). Data are presented with standard deviation. Statistical significances were calculated between WT and TG-33 mice (* *p* ≤ 0.05, ** *p* ≤ 0.01).

We examined the effect of Nef protein expression on heart function. For heart function analysis, 10–13-week-old male and female Nef-transgenic mice and their WT littermates were subjected to transthoracic echocardiography. The analysis of the echocardiography data showed that fractional shortening (FS) was significantly decreased in Nef-transgenic mice compared to their WT littermates (Figure 2A,B, Table 1). Also, the TG-33 mice had significantly decreased left ventricle posterior wall (LVPW) thickness during systole (Table 1).

In contrast, the diameter and volume of the left ventricle were significantly increased in the transgenic mice during both systole and diastole (Figure 2C,D, Table 1). Further, we monitored the heart function of TG-33 mice at 24 weeks and 48 weeks. Our data suggest that Nef expression causes a significant compromise in cardiac function and heart failure at 48 weeks (Figure S2a–f, Table 1). The survival of the TG-33 mice was monitored for 12 months, and we found that Nef-expressing mice have end-stage heart failure and mortality at 45–57 weeks (Figure S3, Table 1). Similar to the TG-33 transgenic mice line, other lines (TG-12, TG-22, and TG-56) also show cardiac dysfunction (Table S1).

### 2.4. Nef Protein Expression Dysregulates Autophagy in Cardiac Tissue

**Figure 3:**
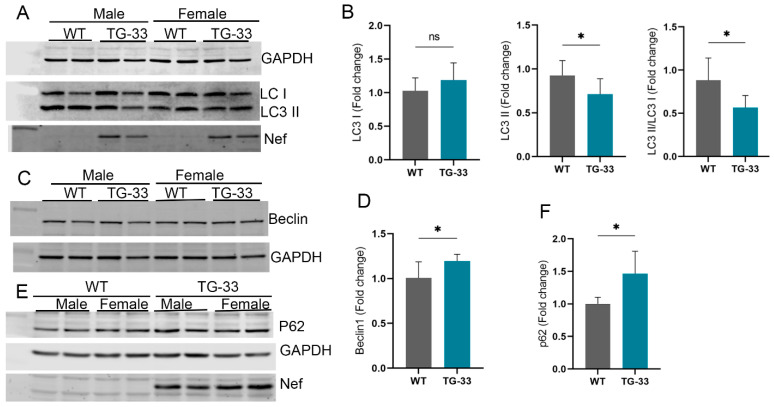
**Nef expression alters autophagic markers in the heart of TG-33 mice.** The Western blots and graphs show expression of autophagy marker proteins (**A**,**B**) LC3, (**C**,**D**) Beclin-1, and (**E**,**F**) p62 in the left ventricular tissue of adult mice hearts (10–13 weeks old) (*n* = 11 WT (6 males, 5 females) and 7 TG-33 (3 males, 4 females)). GAPDH was used as a loading control. Data are presented with standard deviation. Statistical significances were calculated between WT and TG-33 mice (* *p* ≤ 0.05, ns = non-significant).

**Figure 4:**
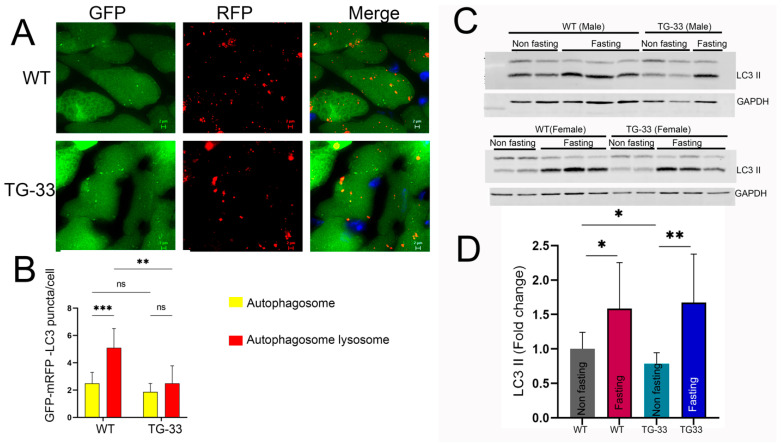
**Nef expression decreases autophagy flux in the hearts of TG-33 mice.** TG-33 mice were crossed with autophagy reporter mice tfL-C3 to monitor the autophagy process in the heart. Age-matched transgenic tfL-C3 mice were used as a control group. (**A**) Representative fluorescence microscopic images show autophagosome (yellow puncta) and autophagosome–lysosome fused (red puncta) formation in the adult mouse tissue of control and TG-33 mice. (**B**) The graph shows the quantification of the mean number of yellow and red puncta per cardiomyocyte (*n* = 7 tfL-C3 (WT) mice (3 males, 4 females) and 6 TG-33 (3 males, 3 females)). (**C**,**D**) Western blot and graph show autophagy of Nef-transgenic mice during non-fasting and fasting conditions (*n* = 10 WT non-fasting mice (4 males, 6 females), 9 WT fasting mice (5 males, 4 females), 10 TG-33 non-fasting mice (5 males, 5 females), and 7 TG-33 fasting mice (3 males, 4 females)). Data are presented with standard deviation. Statistical significances were calculated between WT and TG-33 mice (* *p* ≤ 0.05, ** *p* ≤ 0.01, *** *p* ≤ 0.001, ns = non-significant).

Accumulating evidence indicates that HIV proteins regulate the cellular autophagy process to gain access to the host defense system and successfully complete the replication cycle. Specifically, in vitro studies from our lab and others show that Nef can dysregulate the cellular autophagy process and may lead to cell death. To further understand the effect of Nef protein at the organ level, we detected cellular autophagy in the TG-33 mice heart tissue by Western blotting. Interestingly, we found that autophagy marker protein LC3 II expression was significantly reduced in Nef-transgenic mice compared to their littermates (Figure 3A,B). Further Western blot data show that TG-33 mice hearts have increased accumulation of Beclin-1 (Figure 3C,D) and p62 (Figure 3E,F). We also detected the autophagy flux in the Nef-expressing transgenic mice using autophagy reporter mice tf-LC3 as described earlier [15]. TG-33 mice were crossed with the tf-LC3 mice, and heart tissues were fixed for the cryosection. Thin cryosections of the fixed heart tissue were prepared for the microscopic analysis of the autophagy puncta. Images were captured under a fluorescence microscope, and autophagy puncta were counted in captured images by ImageJ software, version 1.52b (NIH). Our analysis shows that Nef-expressing tissue has a reduced level of autophagy (Figure 4A,B). Furthermore, we also monitor autophagy in the TG-33 mice during autophagy induction by keeping mice in a starving condition. The level of autophagy was detected by Western blotting using LC3 antibody. Our study found that starvation significantly increased the expression of LC3 II in TG-33 mice (Figure 4C,D). These data suggest that starvation-mediated induction of autophagy recovers autophagy in the TG-33 mice.

### 2.5. Nef Protein Dysregulates Autophagy Through Increased Stability of Bcl2

**Figure 5:**
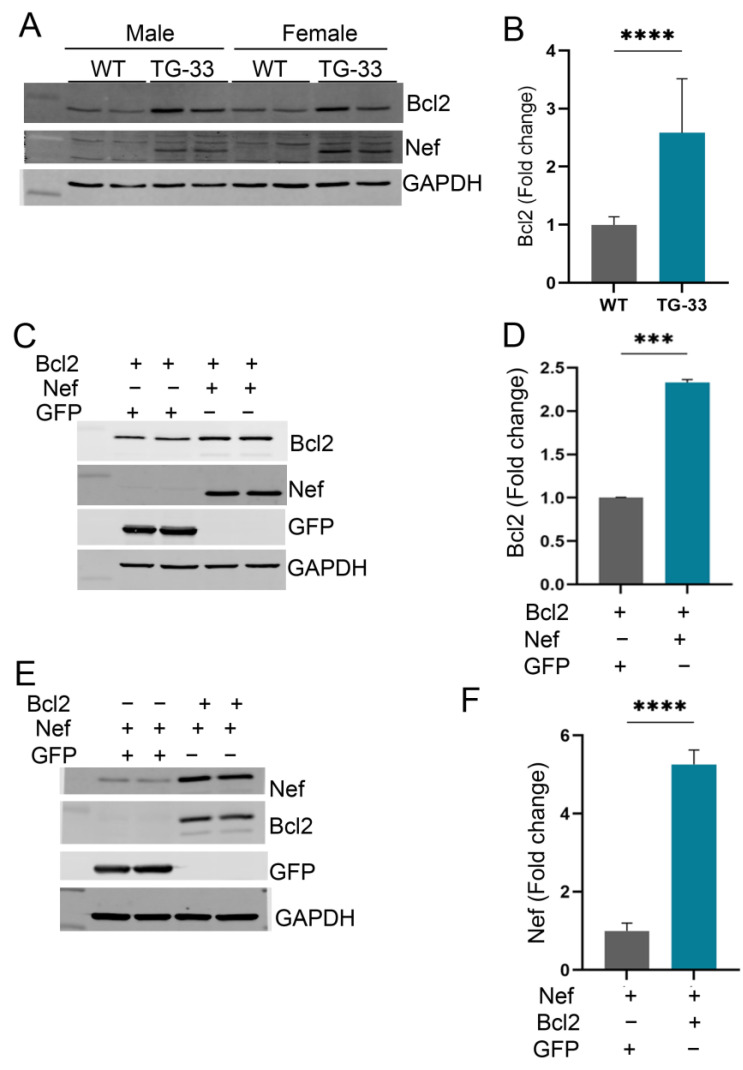
**Nef protein upregulates Bcl2 protein expression in the cardiac tissue of TG-33 mice and HEK293 cells.** (**A**) Western blot shows the expression of Bcl2 protein in the heart tissue. Western blot was performed in cardiac tissue protein lysate of 10 -13-week-old WT and TG-33 mice. GAPDH was used as a loading control. (**B**) The graph shows quantification of Bcl2 protein in the heart (*n*= 11 WT (6 males, 5 females) and 7 TG-33 (3 males, 4 females)). Data are presented with standard deviation. Statistical significances were calculated between WT and Nef-TG mice (**** *p* ≤ 0.0001). Western blots show the expression of (**C**) Bcl2 and (**E**) Nef proteins in HEK293 cells transfected with the Nef and the Bcl2 plasmids. GAPDH was used as a loading control. The graphs show the quantification of (**D**) Bcl2 and (**F**) Nef proteins in HEK293 cells. The experiment was repeated three times. Data are presented with standard deviation. Statistical significances were calculated between the control group and cells expressing both Bcl2 and Nef proteins (*** *p* ≤ 0.001, **** *p* ≤ 0.0001).

Earlier studies from our lab and others show that Nef can interact with autophagy regulator proteins such as Beclin-1 and p62. To elucidate the mechanisms of Nef-mediated inhibition of the autophagy process, we tested the expression of different autophagy regulatory proteins. Interestingly, we found that expression of Bcl2 significantly increased in TG-33 mice heart tissue compared to WT mice (Figure 5A,B). To understand the effect of the Nef protein on the Bcl2 protein expression level, we transfected the Nef plasmid with the Bcl2 plasmid in the HEK293 cells. Our Western blot data show that Nef expression significantly increases the expression of Bcl2 protein in the cells (Figure 5C,D). Additionally, we checked the expression of Nef protein in the presence of Bcl2 by transfection of plasmid DNA in HEK293 cells. Our study found that Bcl2 expression also increases Nef protein expression in the cells (Figure 5E,F).

### 2.6. Nef Protein Induces Senescence-Associated Gene Expression

**Figure 6:**
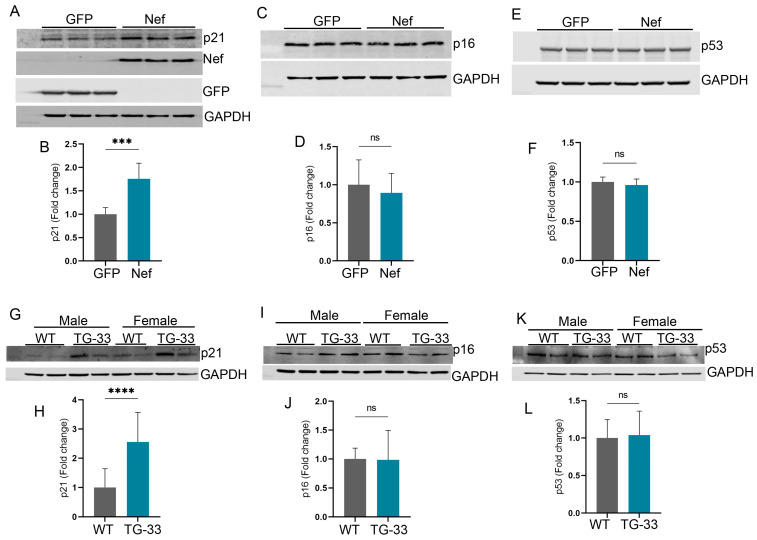
Nef protein expression is associated with increased p21 protein but not p16 and p53 in HEK293 cells and in the heart tissue. The representative Western blots show the expression of (**A**) p21, (**C**) p16, and (**E**) p53 proteins in the HEK293 cells transfected with the Nef plasmid or GFP. GAPDH was used as a loading control. The graphs show the quantification of (**B**) p21, (**D**) p16, and (**F**) p53 proteins in the transfected HEK293 cells. The experiment was repeated three times. The Western blots show the expression of (**G**) p21, (**I**) p16, and (**K**) p53 proteins in the cardiac tissue protein lysate of 10–13-week-old WT and TG-33 mice. GAPDH was used as a loading control. The graphs show the quantification of (**H**) p21, (**J**) p16, and (**L**) p53 proteins in the heart tissue (*n* = 11 WT (6 males, 5 females) and 7 TG-33 (3 males, 4 females)). Data are presented with standard deviation. Statistical significances were calculated between GFP and Nef-transfected HEK293 cells and WT and TG-33 mice (*** *p* ≤ 0.001, **** *p* ≤ 0.0001, ns = non-significant).

Earlier studies suggested that HIV plays a critical role in the regulation of senescence-associated gene expression [16]. To understand the effect of Nef protein in the senescence-associated signaling, we monitored the expression of senescence markers in HEK293 cells by transfection of Nef plasmid and GFP control plasmid. Our study shows that the Nef protein causes significantly increased expression of p21 in Nef-expressing HEK293 cells (Figure 6A,B). However, the expression of other senescence markers, such as p16 and p53, did not change (Figure 6C–F). We have also assessed the expression of senescence-associated protein markers in Nef-transgenic mice hearts (Figure 6G,I,K) by Western blotting. Consistent with the cell culture finding, Nef-transgenic mice hearts have increased expression of p21 (Figure 6H), but the expression of p16 and p53 did not change (Figure 6J,L). Additionally, we performed a SA-βgal assay to check the Nef-mediated induction of senescence phenotype. HeLa cells were transfected with the pcDNA3.1Nef and or pShuttle plasmid for 48 h, and SA-βgal assay was performed in the fixed cells. Our analysis showed that Nef expression significantly induced the senescence phenotype in the cells (Figure S4). To determine the effect of Nef protein in cardiac pathology, we also performed lipofuscin staining in the heart tissue of the TG-33 mice. Lipofuscin is an intracellular aggregate and has been found to be associated with aging [17]. Our study found that the number of lipofuscin puncta significantly increased in the heart of the 24-week-old TG-33 mice compared to their wild-type littermates (Figure S5).

## 3. Discussion

In the past, several studies were conducted to test the effect of HIV on cardiovascular function. Clinical studies suggest that HIV patients develop cardiovascular complications in the early stages of HIV infection [18,19]. Recent studies indicate cardiovascular disease is a significant cause of comorbidity in the HIV population, which causes heart failure and morbidity in the survival population [20]. The application of improved antiretroviral drugs significantly enhances the survival of the HIV population; however, PLWHA show an early sign of premature aging compared to the general population [16,21,22,23]. Several transgenic and humanized mouse models were generated to mimic in vivo HIV infection. Consistent with the clinical data, HIV mouse models that express HIV proteins in various organs also show compromised organ function, including cardiovascular complications [24,25].

Studies performed in nonhuman primate models using Nef deleted SIV Nef (-)SIV show that Nef is an essential virulence factor for HIV [26]. Further in vitro and some in vivo studies suggest that Nef plays an important role in the survival and pathogenesis of HIV [27,28]. Expression analysis of HIV patients suggests that Nef protein can be detected in different organs, including plasma, endothelial cells, immune cells, brain, lung, heart, etc. [8,29,30]. Further study shows that Nef protein plays an important role in the longevity of immune cells and inhibits cell death [31]. However, to examine the effect of individual Nef protein in the development of HIV-mediated pathogenesis, we needed mouse models that express Nef protein.

To test the role of Nef protein at the organ level, we developed a new transgenic mouse that can express Nef proteins in an adult mouse heart. In the past, other laboratories have also developed Nef-transgenic mice to study the effect of Nef in vivo [32]. However, their models are restricted to expressing Nef protein in only one organ. Compared to other limited Nef mouse models, our Nef-transgenic mice are able to express the protein in cells, tissue, and in an organ-specific manner depending on the Cre promoter [33]. Our study shows that Nef-transgenic mice express Nef protein in the heart in the presence of α-MyHC-Cre recombinase.

Accumulating evidence suggests that HIV infection promotes cardiovascular disease, including cardiomyopathy and heart failure in PLWHA [34,35]. Since HIV induces cardiovascular complications, we tested the heart function of newly developed Nef-transgenic mice. Our study shows that Nef protein compromises heart function. Further, we found that Nef protein expression caused cardiac remodeling, increased fibrosis, and cellular hypertrophy. Consistent with our findings, Castro et al. reported that HIV induces ventricular dysfunction in PLWHA through a reduction in left ventricular ejection fraction and leads to heart failure [36]. A past study conducted on the porcine model shows that Nef protein causes endothelial dysfunction and vasorelaxation in pulmonary arteries [37]. Similarly, mouse models that express Nef protein in endothelial cells show vascular dysfunction [29,38]. The effect of Nef was also tested in lung pathogenesis using a macaque model of SIV infection. Interestingly, a chimeric SIV expressing HIV Nef protein causes pathological changes like intimal disruption and medial hypertrophy [39]. Further, we observed that Nef expression in the heart causes cardiac hypertrophy, leading to later-stage dilation. HIV-infected patients show a higher prevalence of fibrosis in the myocardial tissue, which may be distributed focally or diffusely throughout the heart [40]. The increased fibrosis may cause diastolic and systolic dysfunction and heart failure in HIV carriers [41]. Our study also shows higher fibrotic tissue in the interstitial and perivascular regions in Nef-transgenic mice. Further, our study revealed the role of Nef protein in the development of fibrosis in tissue, which may trigger heart failure in HIV-infected patients. Moreover, survival curve analysis shows that mouse expressing Nef protein have premature death due to heart failure by the age of 12 months. In line with our findings, a clinical study conducted for 5 years using 193 HIV-infected children shows a strong association between left ventricular dysfunction and a lower survival rate in the patients [42]. Previous studies also show the effect of other HIV proteins, such as tat and gp120, in the development of fibrosis and inhibition of cardiac function [43,44,45]. Further, studies showed that other cardiotropic viruses, such as Coxsackievirus B, Human Herpesvirus, Epstein–Barr Virus, cytomegalovirus, Varicella-zoster Virus, Parvovirus B19, Influenza virus, and SARS-CoV-2, could damage the heart through inflammation, cell death, and inhibition of electrical conduction of the heart [46].

We have analyzed cellular autophagy-related signaling to understand the molecular changes associated with the Nef protein in the heart. Previous studies showed that the Nef protein dysregulates autophagy and leads to cell death [8,47]. Our study also shows that Nef expression causes autophagy dysregulation. Further autophagy flux analysis shows that Nef causes inhibition of autophagy. Autophagy is an important cell mechanism that participates in cellular protein quality control and the removal of old and damaged subcellular organelles. Dysregulation of autophagy causes the accumulation of aggregated proteins and induces cellular toxicity. Compromised autophagy can also lead to the development of heart failure and premature death [13,48]. Expression of Nef protein induces cellular toxicity and apoptosis. Our study found that Nef-transgenic mice have dysregulation of autophagy and early mortality.

HIV infection manipulates the cellular autophagy system for survival and replication in the host cells. Different HIV proteins show differential effects on autophagy during HIV replication. Inhibition of autophagy in the host cells inhibits the viral replication and production of virus particles. In contrast, HIV inhibits autophagy at the maturation stage to avoid the degradation of mature virus particles. Previous studies suggest that Nef protein expression can be detected at a very early stage of virus replication [49]. Additionally, it was found that Nef protein inhibits autophagy to maximize the virus particle production through inhibition of the maturation stage [49]. Our previous study based on cell culture found that Nef-treated cardiomyocytes show inhibition of autophagy, accumulation of LC3 II, and cell death. Additionally, we found that treatment with an autophagy inducer, such as rapamycin, can restore autophagy in the Nef-expressing cardiomyocytes [8]. In this study, we evaluate the role of the Nef protein in the autophagy of the heart. Interestingly, under in vivo conditions, we noticed inhibition of autophagy and a significantly decreased level of LC3II in the hearts of Nef-transgenic mice. These studies suggest that Nef has different effects at the organ level than at the cellular level. However, consistent with the in vitro study with rapamycin, we noticed that starvation-mediated autophagy induction can restore Nef-transgenic mice’s autophagy. To understand the mechanism of Nef-mediated autophagy regulation, we evaluated the expression of two important proteins, Beclin-1 and Bcl2, in the hearts of Nef-transgenic mice. Interestingly, we found that Beclin-1 and Bcl2 expressions were significantly elevated in the mice expressing Nef protein. Our previous cell culture study found that Nef interacts with Beclin-1 [8]. In this study, we found that Nef regulates the expression of Bcl2 along with Beclin-1. Bcl2 belongs to the large Bcl2 family protein, which determines the cell’s fate of survival or apoptosis depending on the cell type. Bcl2 also inhibits cellular autophagy by modulating the Beclin-1 function. In our study, we found Nef causes increased accumulation of Bcl2 and Beclin-1 in cells and cardiac tissue. Increased expression of Bcl2 may inhibit Beclin-1 function and can suppress the autophagy process. Consistent with our findings, another study also showed that Nef protein causes inhibition of autophagy through increased interaction of Bcl2 and Beclin-1 in THP-1 macrophages and primary CD4^+^ cells [14].

Several studies were conducted to understand the relationship between HIV and Bcl2 in different cell types. Studies suggest that a strong correlation exists between the expression of Bcl2 and HIV infection. Lower expression of Bcl2 in CD8^+^ cells shows higher viral load and cell death [50]. Similarly, during chronic infection, expression of Bcl2 significantly decreased in the HIV-positive CD4^+^ cells [51]. To confirm the relationship between HIV and Bcl2 expression, authors have treated the HIV-infected cells with ART. Interestingly, the expression of Bcl2 significantly increased in ART-treated cells [52]. Similarly, lymphoid tissue, the major site of viral replication and dissemination of HIV, shows suppression of Bcl2 expression [53]. These studies suggest that a decreased level of Bcl2 promotes viral replication and the spread of viral infection. However, contrastingly, HIV-infected macrophages behave differently than HIV-infected CD4 and CD8 cells. In chronically infected patients, macrophages and microglia carry the latent virus [54]. These cells show increased survival and resistance to apoptosis as well as higher levels of Bcl2 [55]. Studies were conducted to establish the expression of Bcl2 and viral latency. Interestingly, a subset of T cells that promote HIV-1 latency expressed a higher level of Bcl2 [55]. These studies suggest that the level of Bcl2 is one of the determining factors of HIV replication and latency. Our study found that Nef protein expression promotes a higher level of Bcl2. Since Bcl2 negatively regulates autophagy, increased Bcl2 expression will cause pathological remodeling in the heart in the long run. Further, our study found that Bcl2 expression significantly upregulates Nef protein expression in cells. Bcl2 is known for inhibiting autophagy, and Nef protein is degraded through autophagy [8]. So, our study suggests that the interplay of Bcl2 and Nef may play a critical role in HIV-infected cells to protect the infected cells from undergoing apoptosis. The current ART therapy failed to eradicate HIV from patients and let the virus hide at a latent stage. However, viral proteins are still producing and causing comorbidities. Thus, there is a need to develop a latent model to understand the pathogenesis and treatment of HIV. Humanized mice models were generated to understand the pathogenesis of HIV. However, these mice are not suitable for understanding the latency due to a lack of proper HIV-induced latency [56]. Nef proteins can be detected in PLWHA undergoing ART therapy [5,7,8]. Our mice model can express Nef protein at the adult stage and induce latency factors. This mice model could be useful to understand the HIV-induced latency in different organs.

HIV-positive patients show comorbidities and accelerated aging even during ART treatment. In this study, we tested the potential of Nef protein in the development of aging. HIV-1 proteins regulate the expression of several senescence factors in the cells and promote or accelerate cellular apoptosis depending on the cell type or stage of viral replication [23]. In our study, we found that Nef protein significantly enhances the expression of p21 in vitro as well as in cardiac tissue. This protein plays a significant role in the cell cycle progression. Studies suggest that p21 suppresses HIV replication by reducing the intracellular dNTP pools [57]. However, p21 also causes cell cycle arrest and promotes senescence [58,59]. Our study found that both Bcl2 and p21 were significantly upregulated in cardiac tissue. Additionally, our study found that the level of SA-βgal staining significantly increased due to Nef expression in the cells. Furthermore, we found that Nef-transgenic mice accumulate higher amounts of lipofuscin in the cardiomyocyte compared to their wild-type littermates. The higher SA-βgal staining and increased lipofuscin are typical hallmarks of aging [17,60]. These molecules may play a critical role in Nef-mediated senescence and the development of aging in PLWHA. Autophagy plays an important role in the regulation of senescence [61]. Studies suggest that inhibition of autophagy causes the accumulation of damaged organelles and the production of ROS, which causes DNA damage and upregulation of senescence [62]. Our study found that Nef dysregulates autophagy through upregulation of Bcl2, accumulation of Beclin-1, and induction of senescence marker p21. The complex interaction of Nef with other cellular proteins triggers the senescence and induction of aging-like phenotypes.

In conclusion, we have generated a transgenic mouse that can express Nef protein using a Cre-mediated recombination system in adult hearts. Further, this mouse model demonstrates that Nef protein can inhibit organ function and promote heart failure and premature death in the mouse. In addition, we found that Nef protein causes cellular protein quality control dysregulation like autophagy. Further, we found that Nef expression causes the accumulation of two autophagy regulator proteins, Bcl2 and Beclin-1. This association and accumulation of these proteins may trigger a pathological remodeling in the heart through the dysregulation of autophagy. Further, our study shows that adult Nef-transgenic mice generate senescence markers in the heart. Therefore, this mouse model can be helpful in studying the effect of Nef protein in cells, tissue, organs, and host physiology. Additionally, Nef-transgenic mice will help to understand the role of Nef protein in the survival of HIV at the organ levels and generate a better cure for latent HIV for mankind.

## 4. Materials and Methods

### 4.1. Cell Culture and Plasmid Transfection

HEK293 cells were used to test the effect of Nef protein on cellular signaling. Cells were grown in high-glucose DMEM media with 10% FBS and 1X penicillin and streptomycin. Plasmid DNAs (pcDNA 3.1 Nef SF2, pcDNA 3.1 Bcl2, pEGFP) were transfected using Lipofectamine 2000 (Life Technology, Waltham, MA, USA). Transfected cells were grown for 48 h in DMEM media with 2% FBS. Cells were harvested for total protein isolation in RIPA buffer.

### 4.2. Animal Model and Ethics

Animals were handled in accordance with the guidelines provided by the *Guide for the Care and Use of Laboratory Animals*. All the experiments performed in this study were approved by the Institutional Animal Care and Use Committee of the University of Central Florida. Mouse colonies were maintained on a 12 h light and dark cycle in ventilated animal research cages with a chow diet. For the generation of Nef-transgenic mice, the Nef ORF was amplified by using plasmid pcDNA 3.1 Nef SF2 (NIH AIDS reagents) and cloned in a CAG-Lox-CAT vector [63]. Transgenic mice were generated with the help of Cyagen laboratory (Santa Clara, CA, USA). Several positive founder mice were received and crossed with the alpha myosin heavy chain promoter Cre mice (α-MyHC-Cre) to express Nef protein in the heart. Most experiments were performed with age- and gender-matched Nef-transgenic and nontransgenic littermates.

### 4.3. Heart Function Analysis by Echocardiography

Heart function was analyzed in adult male and female mice at 10–13 weeks of age using Visual Sonics Vevo 3100 (FujiFilm VisualSonic, Toronto, ON, Canada). Mice were anesthetized with 3% isoflurane and further maintained with 1.5% isoflurane. Images were captured on the parasternal short-axis view of the left ventricle (LV) at the mid-papillary level [64]. M-mode was used to assess the echocardiography parameters. Left ventricular ejection fraction (EF), fractional shortening (FS), cardiac output (CO), and stroke volume (SV) were calculated using Vevo LAB software (version 5.6.1) [65].

### 4.4. Western Blot Assay

For Western blot analysis, heart tissues were collected from adult mice. For the preparation of total protein lysate, 25 mg of the heart tissue was minced with scissors and homogenized in RIPA buffer (50 mM Tris-HCl pH-8.0, 150 mM NaCl, 1% IGEPAL, 12 mM sodium deoxycholate, 0.1% SDS) with protease inhibitor cocktail (Sigma, St. Louis, MO, USA) by using Bead Mill 24 Homogenizer (ThermoFisher Scientific, Waltham, MA, USA). Homogenates were centrifuged to remove cell debris at 5000 rpm for 5 min and then at 12,000 rpm for 30 min at 4 °C, and supernatants were collected for Western blotting [64]. For the preparation of proteins from HEK293 cells, cells were washed twice with 5 mL of 1X PBS, and 500 µL of RIPA buffer with protease inhibitor was added to each plate. Plates were incubated on ice for 15 min, and lysed cells were collected in 1.5 mL centrifuge tubes. The cell lysate was vortexed and centrifuged at 10,000 rpm for 10 min at 4 °C. The supernatants were collected for the Western blot. Protein concentration was determined by Bradford protein assay (Sigma). For Western blotting, proteins were separated in sodium dodecyl sulfate-polyacrylamide gel (SDS-PAGE) (Bio-Rad, Hercules, CA, USA). Then, they were transferred to polyvinylidene difluoride (PVDF) membranes (Bio-Rad) by wet transfer or semi-dry Trans-Blot Turbo System (Bio-Rad). After transfer, membranes were incubated in blocking buffer (Li-COR, Lincoln, NE, USA) for 2 h and then probed with primary antibodies in blocking buffer at 4 °C overnight. Following primary antibody incubation, membranes were washed twice with 1X PBST and once with 1X PBS. The membranes were exposed to secondary antibodies labeled with IRDye for 2 h at room temperature in the antibody dilution buffer (Li-COR). Membranes were rewashed with 1X PBST and 1X PBS and scanned using a Li-COR Odyssey Infrared Imaging System (Li-COR) [66]. The blots were quantified by using Image Studio software (ver. 4.0, Li-COR). The following primary antibodies were used for the Western blotting: HIV-1 Nef (AIDS reagent), LC3 (Sigma), Beclin-1, Bcl2, p16, p21, p53, GAPDH (Proteintech Group, Rosemont, IL, USA), and p62/SQSTM1 (Novus Biological, Centennial, CO, USA). GAPDH was used as a loading control in most of the experiments.

### 4.5. Histology

For the histological study, 10–13-week-old mice were anesthetized with isoflurane, and their hearts were excised after perfusion in 10% Neutral Buffered Formalin (NBF) (Epredia, Kalamazoo, MI). The isolated hearts were cut longitudinally and fixed in 10% NBF (Epredia) overnight at room temperature. The fixed heart tissues were processed using a spin tissue processor STP 120-2 (Epredia) and paraffin-embedded using a tissue embedding station (LEICA, Deer Park, IL, USA). Paraffin blocks with embedded heart tissue were sectioned at 5 µM thickness using Microtome (LEICA). For histological analysis, heart tissue sections were deparaffinized and stained with hematoxylin and eosin (H&E) (Sigma) and Masson’s trichrome staining (Sigma) as described before [67]. Images were captured using a BZ-X800 Keyence microscope (Keyence, Osaka, Japan) with a 2 × objective for H&E-stained sections and a 20× objective for Masson’s trichrome-stained sections. Masson’s trichrome-stained images were analyzed using BZ-X800 analyzer software, version 1.1.1.8. The staining of wheat germ agglutinin (WGA) (Vector Laboratories, Burlingame, CA, USA) was performed according to the manufacturer’s protocol. The slides were baked at 60 °C for 2 h, deparaffinized, and rehydrated as follows: two times for 10 min in Xylene, three times for 4 min in 100% ethanol, three times for 4 min in 95% ethanol, and three times for 3 min in distilled water. After antigen retrieval with 1× citrate buffer (Vector Laboratories, Vernon Hills, IL, USA), the slides were washed with 1× PBS and incubated with blocking buffer (5% BSA, 0.1% Tween 20, 1× PBS) for 1 h at room temperature. After removing the blocking buffer, sections were incubated in 2 ug/mL WGA (Vector Laboratories) for 30 min and then washed twice with 1× PBST and once with 1× PBS. Then, the sections were incubated in 1:5000 diluted DAPI (ThermoFisher Scientific) for nuclear staining at room temperature for 5 min and washed three times with 1× PBS. An antifade mounting medium with phalloidin (Vector Laboratories) was used to mount tissue on a slide. Images were captured using a BZ-X800 Keyence microscope (Keyence). Cell areas were calculated using ImageJ software, version 1.52b (National Institutes of Health (NIH), Bethesda, MD, USA).

### 4.6. Autophagy Assay

Autophagy in cardiac tissue was determined by Western blotting with the LC3 (Sigma) antibody using total protein lysate. We also used autophagy-reported mice tf-LC3 to detect autophagy in the heart tissue [8]. Nef mice were crossed with the tf-LC3 mice, and heart tissue was fixed for the cryosection as described before [8]. Cryosections were mounted using an antifade mounting medium with DAPI (Vector Laboratories), and images were captured using a BZ-X800 Keyence microscope (Keyence) with a 60× objective [8]. Red and yellow puncta in images were counted manually to determine autophagy flux. Additionally, autophagy in the cardiac tissue was determined during fasting conditions [68]. Mice were starved for 12 h with water and no food. Heart tissues were collected, and autophagy was determined by Western blot using an LC3 antibody (Sigma).

### 4.7. Determination of Lipofuscin

To determine the accumulation of lipofuscin in the cardiac tissue, paraffin sections of the 12-week and 24-week-old mice heart tissue were deparaffinized and hydrated, as described before [23]. Tissue sections were mounted on the slide using antifade mounting media with DAPI (Vector Laboratories). Autofluorescence of the tissue section was captured at 545/610 excitation/emission wavelength using a Keyence microscope (Keyence). Captured images were analyzed by ImageJ software.

### 4.8. SA-βgal Staining

For the SA-βgal assay, HeLa cells were seeded into a 35 mm glass-bottom cell culture dish (Cellvis, Sunnyvale, CA, USA). The next day, cells were transfected with pcDNA 3.1 plasmid expressing Nef and pShuttle plasmid (control) DNA using lipofectamine transfection reagents, and cells were grown to DMEM media having 10% FBS for another 48 h. SA-βgal staining was performed according to the manufacturer’s protocol (Cell Signaling). In brief, cells were washed twice with 1× PBS and fixed with the fixative solution for 12 min. After the fixative, cells were washed with the 1XPBS and incubated with 1 mL of SA-βgal staining solution for 48 h. Images were captured after 48 h using a Keyence microscope (Keyence). The percentage of SA-βgal-positive cells was counted manually using ImageJ software, version 1.52b (NIH).

### 4.9. Statistical Analysis

Data were analyzed using GraphPad Prism software (version 9.4). An unpaired *t*-test and one-way ANOVA were used to determine the difference between the means of the control and experimental groups. The difference was considered statistically significant with a *p*-value ≤ 0.05.

## Figures and Tables

**Table 1:** **Echocardiographic analyses of TG-33 mice.** LV DIAMs, systolic left ventricular diameter; LV DIAMd, diastolic left ventricular diameter; LV VOLs, left ventricular systolic volume; LV VOLd, left ventricular diastolic volume; SV, stroke volume; EF, ejection fraction; FS, fractional shortening; CO, cardiac output; LV mass, left ventricular mass; LVAWs, systolic left ventricular anterior wall thickness; LVAWd, diastolic left ventricular anterior wall thickness; LVPWs, systolic left ventricular posterior wall thickness; LVPWd, diastolic left ventricular posterior wall thickness; HR, heart rate. Data are presented with standard deviation. Statistical significances were calculated between WT and TG-33 mice within the same age group (* *p* ≤ 0.05, ** *p* ≤ 0.01, *** *p* ≤ 0.001, **** *p* ≤ 0.0001).

	WT (12-Week-Old) (*n* = 32)	TG-33 (12-Week-Old) (*n* = 24)	WT (24-Week-Old) (*n* = 30)	TG-33 (24-Week-Old) (*n* = 15)	WT (48-Week-Old) (*n* = 9)	TG-33 (48-Week-Old) (*n* = 11)
LV DIAMs (mm)	2.306 ± 0.4971	2.653 ± 0.3155 **	2.846 ± 0.4455	3.129 ± 0.4737	3.056 ± 0.444	4.634 ± 0.9447 ***
LV DIAMd (mm)	3.66 ± 0.3538	3.857 ± 0.3135 *	3.964 ± 0.3477	4.088 ± 0.4108	4.122 ± 0.31.56	5.133 ± 0.7494 **
LV VOLs (μL)	19.71 ± 9.932	26.49 ± 8.082 **	31.99 ± 11.91	40.15 ± 14.42 *	37.79 ± 14.47	104.5 ± 50.28 **
LV VOLd (μL)	57.45 ± 13.11	64.84 ± 12.7 *	69.29 ± 14.05	74.77 ± 17.65	75.76 ± 14.05	129.2 ± 46.03 **
SV (μL)	37.74 ± 6.044	42.75 ± 10.73 *	37.3 ± 6.426	34.62 ± 4.637	37.98 ± 8.226	24.71 ± 9.025 **
EF (%)	67.45 ± 10.93	59.6 ± 6.322 **	55.08 ± 9.748	47.7 ± 7.655 *	51.02 ± 10.71	22.03 ± 12.49 ****
FS (%)	37.58 ± 8.712	31.32 ± 4.239 **	28.54 ± 6.21	23.8 ± 4.505 *	26.04 ± 6.528	10.32 ± 6.198 ****
CO (mL/min)	16.32 ± 2.956	17.29 ± 3.29	16.98 ± 3.183	15.41 ± 2.299	18.06 ± 3.676	11.31 ± 4.315 **
LV mass (mg)	118.1 ± 22.49	123.6 ± 27.6	136.1 ± 29.2	137.6 ± 24.48	147.2 ± 14.46	197.8 ± 44.18 **
LVAWs (mm)	1.406 ± 0.182	1.319 ± 0.2129	1.36 ± 0.155	1.269 ± 0.2072	1.343 ± 0.1427	1.15 ± 0.2266 *
LVAWd (mm)	0.9511 ± 0.1362	0.9037 ± 0.1623	0.966 ± 0.1217	0.9168 ± 0.1335	0.9776 ± 0.08788	0.9325 ± 0.1545
LVPWs (mm)	1.279 ± 0.2415	1.126 ± 0.1454 **	1.144 ± 0.1769	1.068 ± 0.1257	1.161 ± 0.2137	0.8678 ± 0.1551 **
LVPWd (mm)	0.8287 ± 0.1877	0.816 ± 0.0982	0.8208 ± 0.1386	0.8278 ± 0.1008	0.8435 ± 0.1178	0.7925 ± 0.1315
HR (bpm)	432 ± 29.98	451.8 ± 29.6 *	454.4 ± 18.41	445.8 ± 36.76	577.4 ± 37.72	456 ± 46.34

## Data Availability

The data associated with the work are available upon request to the corresponding author.

## Supplementary Materials

The following supporting information can be downloaded at: https://www.mdpi.com/article/10.3390/ijms252111401/s1.

## Abbreviations

ART	Antiretroviral therapy
CD	Clusters of differentiation
CO	Cardiac output
DAPI	4′,6-diamidino-2-phenylindole
DIAMd	Diastolic left ventricular diameter
dNTP	Deoxynucleotide triphosphate
EF	Ejection fraction
FS	Fractional shortening
H&E	Hematoxylin and eosin
HEK293	Human embryonic kidney 293 cells
HIV	Human immunodeficiency virus
HR	Heart rate
LV mass	Left ventricular mass
LV VOLd	Left ventricular diastolic volume
LV VOLs	Left ventricular systolic volume
LVAWd	Diastolic left ventricular anterior wall thickness
LVAWs	Systolic left ventricular anterior wall thickness
LVPW	Left ventricular posterior wall
LVPWd	Diastolic left ventricular posterior wall thickness
LVPWs	Systolic left ventricular posterior wall thickness
NBF	Neutral Buffered Formalin
ORF	Open Reading Frame
PBS	Phosphate-buffered saline
PBST	Phosphate-buffered saline tween 20
PLWHA	People living with HIV/AIDS
PVDF	Polyvinylidene fluoride or polyvinylidene difluoride
RIPA	Radioimmunoprecipitation Assay buffer
SDS-PAGE	Sodium dodecyl-sulfate polyacrylamide gel electrophoresis
SA-βgal	Senescence-associated β-galactosidase
SIV	Simian immunodeficiency virus
SV	Stroke volume
TG	Transgenic
THP-1	Tohoku Hospital Pediatrics-1
WGA	Wheat germ agglutinin
WT	Wild type
α-MyHC-Cre	Alpha myosin heavy chain promoter Cre
