# Supplementary material for: HIV Protein Nef Induces Cardiomyopathy Through Induction of Bcl2 and p21
Source: Int J Mol Sci. 2024 Oct 23;25(21):11401. doi: 10.3390/ijms252111401 (PMC11547003; doi:10.3390/ijms252111401)
Supplement: Supplementary file 1 [file ijms-25-11401-s001.zip › ijms-3207818-supplementary.pdf]

Figure and legends:

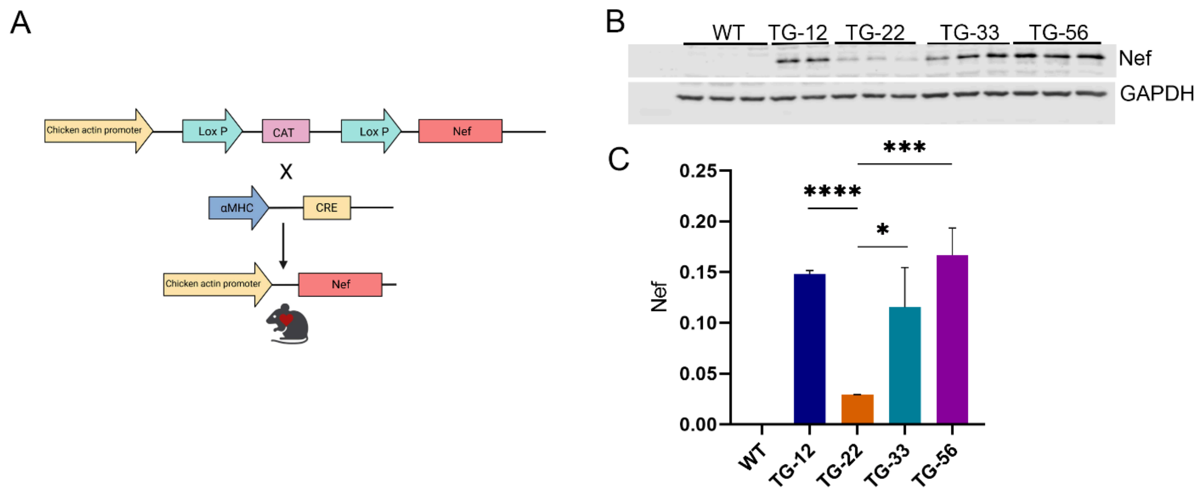

Figure S1. Generation and validation of the Nef transgenic mouse lines. (**A**) The schematic diagram shows the generation of the Nef transgenic (Nef-TG) mouse. HIV-Nef gene was cloned in the CAG-Lox-CAT vector. Four different founder mouse lines were generated. Founder mouse lines were bred with the wild-type mice and crossed with the alpha myosin heavy chain Cre (αMHC-Cre) mice to express Nef protein in the heart. (**B**) Western blot shows Nef protein expression in the heart of Nef-TG lines. GAPDH was used as a loading control. (**C**) The graph shows Nef protein quantification in Nef-TG mice's heart tissue. Data are presented with standard deviation. Statistical significances were calculated between the Nef TG mice lines (*$p \leq 0.05$, *** $p \leq 0.001$, **** $p \leq 0.0001$).

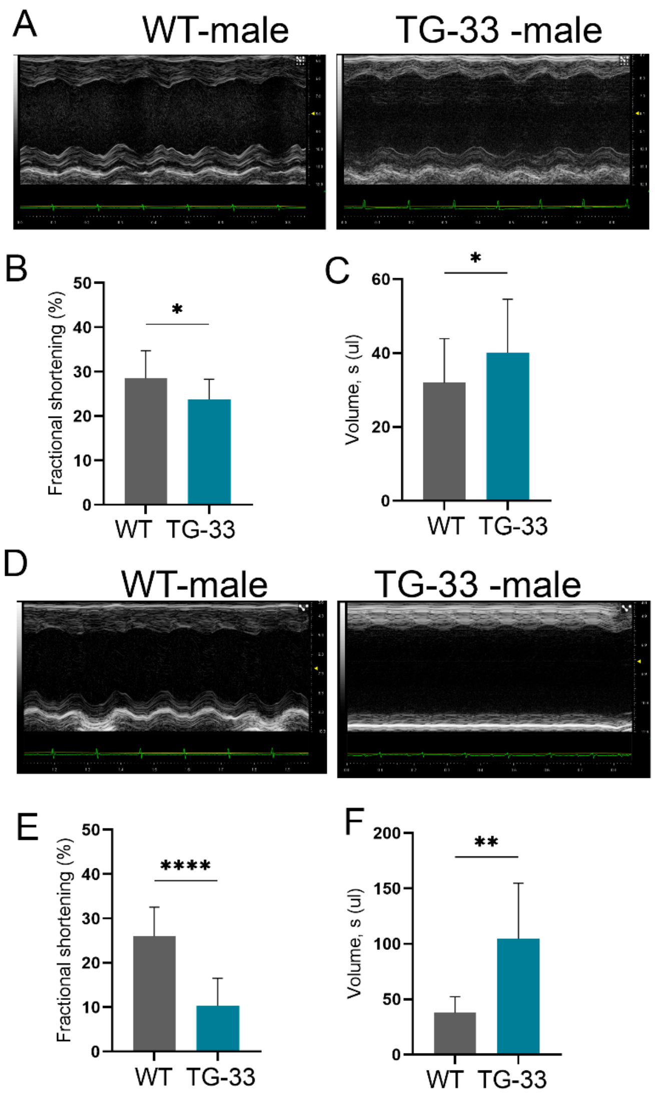

Figure S2. Nef transgenic mice exhibit cardiac dysfunction at 24 weeks of age and heart failure at 48 weeks of age. Representative images show M-mode echocardiography of WT and TG-33 mice at (**A**) 24 and (**D**) 48 weeks of age. The images of the left ventricle were captured at the mid-papillary level (the parasternal short-axis view). Graphs show quantification of (**B**) fractional shortening and (**C**) volume of the left ventricle during systole (volume, s) of 24-week-old mice (n=30 WT (17 males, 13 females) and, 15 TG-33 (6 males, 9 females)), and (**E**) fractional shortening and (**F**) volume of the left ventricle during systole (volume, s) of 48-week-old mice (n=9 WT (7 males, 2 females) and 11 TG-33 (6 males, 5 females)). Data are presented with standard deviation. Statistical significances were calculated between WT and TG-33 mice (*$p \leq 0.05$, **$p \leq 0.01$, **** $p \leq 0.0001$).

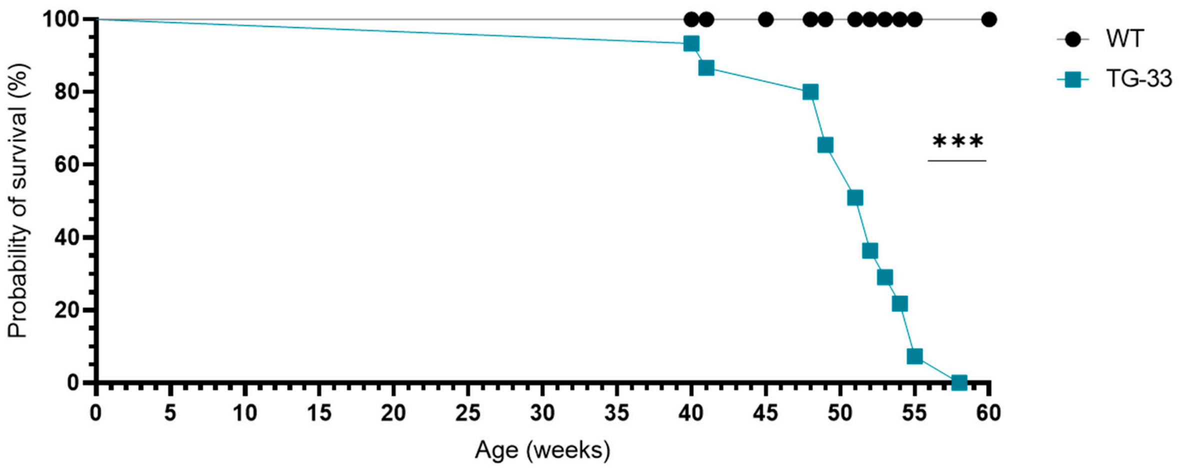

Figure S3. Nef transgenic mice have a shortened life span. Kaplan-Meier survival analysis shows that TG-33 mice have significantly shorter life spans than WT mice. (n=15 WT (7 males, 8 females) and 14 TG-33 (5 males, 9 females)). Statistical significance was calculated between WT and TG-33 mice (*** $p \leq 0.001$).

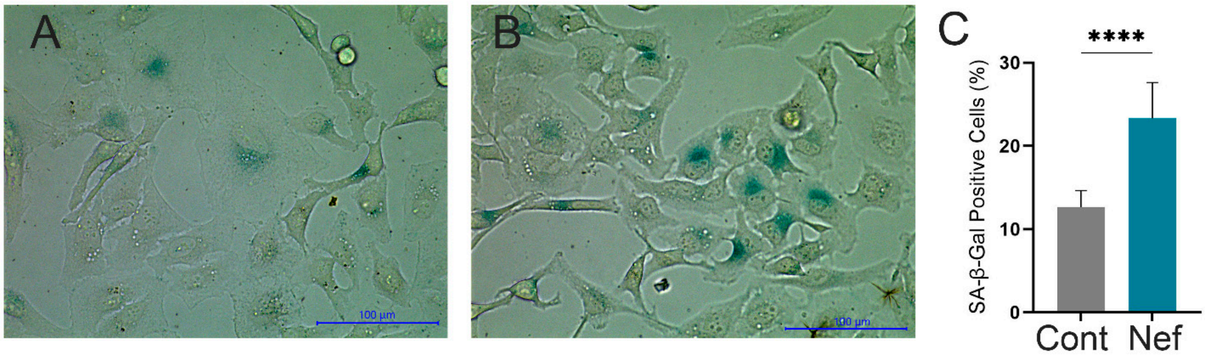

Figure S4: Representative images show that Nef induces senescence in the cells. (**A-B**) HeLa cells were transfected with plasmid DNA for 48 hours and then SA-βgal assay was performed. Cells were incubated in the staining solution for 48 hours and images were captured. (**C**) The graph shows the quantification of the SA-βgal positive cells (n=844 pShuttle cells and 849 Nef expressing cells). Experiments were repeated three times. Data are presented with standard deviation. Statistical significance was calculated between control and Nef expressing cells (**** $p \leq 0.0001$).

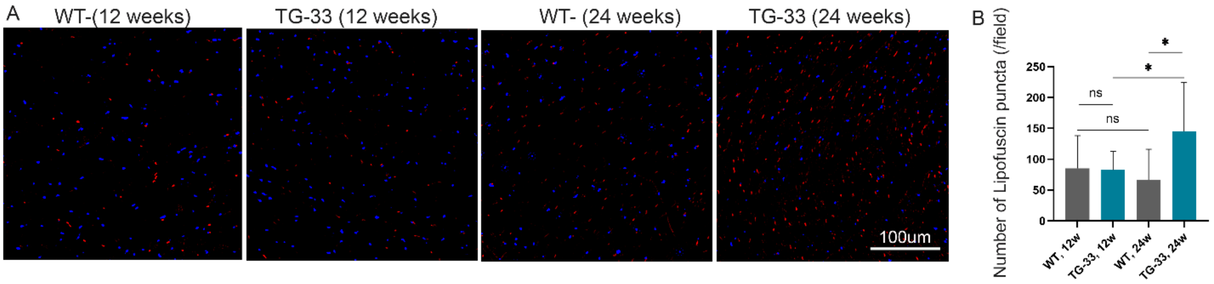

Figure S5: **Representative microscopic images show that Nef causes accumulation of lipofuscin in the heart.** (**A**) Lipofuscin detection was performed in paraffin cardiac tissue sections by measuring the autofluorescence. DNA was stained with DAPI. (**B**) The graph shows counting of lipofuscin positive puncta (n=16 of 12-week-old WT mice (8 males, 8 females), 10 of 12-week-old TG-33 mice (4 males, 6 females), 11 of 24-week-old WT mice (7 males, 4 females), and 12 of 24-week-old TG-33 mice (4 males, 8 females)). Data are presented with standard deviation. Statistical significances were calculated between WT and TG-33 mice in different age group (*$p \leq 0.05$).

	WT (n=32)	Nef TG 12 (n=9)	Nef TG 22 (n=10)	Nef TG 33 (n=24)	Nef TG 56 (n=6)
LV DIAMs (mm)	2.306±0.4971	2.572±0.6309	2.61±0.531	2.653±0.3155**	2.877±0.5356**
LV DIAMd (mm)	3.66±0.3538	3.685±0.5286	3.71±0.4953	3.857±0.3135*	3.925±0.3889
LV VOLs (μl)	19.71±9.932	26.2±16.2	26.44±11.3	26.49±8.082**	33.15±15.91**
LV VOLd (μl)	57.45±13.11	59.25±20.62	60±17.65	64.84±12.7*	67.78±16.25
SV (ul)	37.74±6.044	33.05±5.874*	33.56±8.148	42.75±10.73*	34.64±5.039
EF (%)	67.45±10.93	58.87±11.24*	57.78±10.15*	59.6±6.322**	53.02±11.05**
FS (%)	37.58±8.712	31±7.342*	30.25±6.927*	31.32±4.239**	27.17±6.551**
CO (ml/min)	16.32±2.956	15.03±2.734	13.89±4.204*	17.29±3.29	15.4±1.143
LV mass (mg)	118.1±22.49	121.8±22.26	133.3±21.3	123.6±27.6	143.7±26.91*
LVAWs (mm)	1.406±0.182	1.264±0.1788*	1.451±0.2017	1.319±0.2129	1.372±0.3011
LVAWd (mm)	0.9511±0.1362	0.8596±0.1148	1.002±0.1492	0.9037±0.1623	1.002±0.2632
LVPWs (mm)	1.279±0.2415	1.325±0.32	1.191±0.201	1.126±0.1454**	1.181±0.1246
LVPWd (mm)	0.8287±0.1877	0.9504±0.2286	0.9107±0.1741	0.816±0.0982	0.8838±0.06725
HR (bpm)	432±29.98	456.8±49.99	409.8±49.41	451.8±29.6*	449.1±43.52

Table S1. Echocardiographic analyses of Nef transgenic mouse lines at 12 weeks of age. LV DIAMs, systolic left ventricular diameter; LV DIAMd, diastolic left ventricular diameter; LV VOLs, left ventricular systolic volume; LV VOLd, left ventricular diastolic volume; SV, stroke volume; EF, ejection fraction; FS, fractional shortening; CO, cardiac output; LV mass, left ventricular mass; LVAWs, systolic left ventricular anterior wall thickness; LVAWd, diastolic left ventricular anterior wall thickness; LVPWs, systolic left ventricular posterior wall thickness; LVPWd, diastolic left ventricular posterior wall thickness; HR, heart rate. Data are presented with standard deviation. Statistical significances were calculated between WT and Nef TG mice (*p ≤ 0.05, **p ≤ 0.01).
